# Exploring the pathogenesis of diabetic kidney disease by microarray data analysis

**DOI:** 10.3389/fphar.2022.932205

**Published:** 2022-08-17

**Authors:** Haiyan Cao, Xiaosheng Rao, Junya Jia, Tiekun Yan, Dong Li

**Affiliations:** ^1^ Department of Nephrology, Tianjin Medical University General Hospital, Tianjin, China; ^2^ Department of Urology, The First Affiliated Hospital of Guangzhou Medical University, Guangzhou, China

**Keywords:** diabetic kidney disease, differentially expressed genes, hub genes, microarray data analysis, pathogenesis

## Abstract

Diabetic kidney disease (DKD) is a major complication of diabetes mellitus, and the leading contributor of end-stage renal disease. Hence, insights into the molecular pathogenesis of DKD are urgently needed. The purpose of this article is to reveal the molecular mechanisms underlying the pathogenesis of DKD. The microarray datasets of GSE30528 and GSE30529 were downloaded from the NCBI Gene Expression Omnibus (GEO) database to identify the common differentially expressed genes (DEGs) between the glomerular DKD (GDKD) and tubular DKD (TDKD), respectively. Gene Ontology (GO) and Kyoto Encyclopedia of Genes and Genomes (KEGG) pathway analysis were performed to analyze the function and pathways of the common DEGs. After constructing the protein–protein interaction (PPI) network and subnetwork analysis, three types of analyses were performed, namely, identification of hub genes, analysis of the coexpressed network, and exploration of transcription factors (TFs). Totally, 348 and 463 DEGs were identified in GDKD and TDKD, respectively. Then, 66 common DEGs (63 upregulated DEGs and three downregulated DEGs) were obtained in DKD patients. GO and KEGG pathway analyses revealed the importance of inflammation response, immune-related pathways, and extracellular matrix-related pathways, especially chemokines and cytokines, in DKD. Fifteen hub genes from the 66 common DEGs, namely, IL10RA, IRF8, LY86, C1QA, C1QB, CD53, CD1C, CTSS, CCR2, CD163, CCL5, CD48, RNASE6, CD52, and CD2 were identified. In summary, through the microarray data analysis, the common functions and hub genes greatly contribute to the elucidation of the molecular pathogenesis associated with DKD.

## Introduction

Diabetic kidney disease (DKD), also known as diabetic nephropathy (DN), is a common complication of diabetes mellitus (DM) and the leading cause of chronic kidney disease (CKD) and end-stage renal disease (ESRD) worldwide (Bell et al., 2015; Collins et al., 2015). Approximately, 30% of the patients with type 1 diabetes mellitus and 40% of the patients with type 2 diabetes mellitus develop DKD, with half of them progressing to ESRD (Alicic et al., 2017). Diabetes has become a global health concern worldwide due to obesity epidemic (Chobot et al., 2018). The incidence rate of CKD caused by diabetes was higher than that of glomerulonephritis (Zhang et al., 2016). Meanwhile, the prevalence and associated burden of DKD is increasing in parallel with the incidence of DM. This poses significant socio-economic challenges on the individual, family, and healthcare (Lozano et al., 2012).

Based on the anatomically distinct regions of kidney biopsy samples, DKD can be divided into glomerular DKD (GDKD) and tubular DKD (TDKD). The common characteristics of GDKD involve damaged glomerular filtration barrier, mesangial cell proliferation, and glomerulosclerosis. The major manifestations of TDKD include dysfunction of renal tubular reabsorption and secretion, and tubulointerstitial fibrosis (López-Novoa et al., 2011; Liyanage et al., 2015). The mechanisms of glomerular and tubulointerstitial injury in DKD are different, but there are many intersections of the related pathways and mediators. Hyperglycemic-induced oxidative stress can directly damage renal cells of glomerular, thus leading to tubulointerstitial fibrosis and proteinuria of DKD (Duni et al., 2019; Samsu 2021). In addition, current studies have also revealed that the complement system is acknowledged as a key factor in the development of glomerular and tubulointerstitial lesions in DKD. For example, the deposition of (complement 5) C5a is not only involved in the morphological changes of the glomerular but also mediates the tubulointerstitial injury in DKD (Tan et al., 2020; Budge et al., 2021). Extensive evidence reported that the interaction between the glomerular and tubulointerstitial damages further contribute to high risk of occurrence and progression of DKD (Arora and Singh 2013). Once DKD triggers the chronic progression of glomerular or tubular damage, the renal lesions of DKD patients are irreversible and progressive. Thus, there is an urgent need to introduce methods that identify the cases of DKD and provide possibilities of underlying causes as well as promising opportunities for treating DKD.

DKD has been traditionally characterized by the interaction of hemodynamic and metabolic factors, including increased systemic and intraglomerular pressure, as well as the subsequent modifications induced by high glucose (Anders et al., 2018). Current evidence indicates that DKD is a multifactorial disease, affected by both genetic and environmental factors. The complex pathogenesis of DKD makes its accurate and early diagnosis difficult. DKD is clinically diagnosed by detecting macroalbuminuria in a diabetic patient. However, some studies have shown that the clinical biomarker of DKD (albuminuria) is variable, since some patients progress to DKD without the occurrence of significant proteinuria (Vaidya et al., 2011; Mottl et al., 2013). In a survey of scientific databases, the number of reported biomarkers indicating DKD (glomerular and tubular damage markers) has increased. These biomarkers include nonalbumin protein-to-creatinine ratio, kidney injury molecule 1 (KIM-1), laminin, and orosomucoid (Papadopoulou-Marketou et al., 2017). However, only a few of these biomarkers are available for routine clinical application. Under such conditions, identifying new diagnostic and prognostic biomarkers and recognizing whether these alternations predict clinical outcomes seems necessary. Fortunately, high-throughput screening techniques provide an opportunity to achieve these goals.

Common transcription features can enhance our understanding of the pathogenesis of glomerular and tubulointerstitial injury in DKD. The purpose of the present study is to explore the hub genes associated with the shared mechanisms of glomerular and tubulointerstitial injury in DKD. We analyzed the gene expression profiles of GDKD (GSE30528) and TDKD (GSE30529) to identify the common differentially expressed genes (DEGs), and further examined the functions and pathways. A constructed protein–protein interaction (PPI) network was used to recognize subnetworks and hub genes. Finally, we obtained 15 hub genes and explored the transcription factors (TFs) that drive the progression of DKD by the microarray data analysis. Taken together, the identified hub genes and enriched pathways between the glomerular and tubulointerstitial lesions offer the possibilities for understanding a common etiology and pathological mechanism of DKD.

## Materials and methods

### Data acquisition and principal component analysis

We acquired gene expression profiles from the Gene Expression Omnibus (GEO) database through the keyword “DKD” or “DN” (Barrett et al., 2013). The inclusion criteria were as follows: 1) dataset from the same sequencing platform that covered the maximum sample sizes and 2) the studied objects should come from the microdissected kidneys of *Homo sapiens*. We downloaded and analyzed the gene expression profiles of GDKD (GSE30528) and TDKD (GSE30529) from the GPL571 platform [Affymetrix Human Genome U133A 2.0 Array (HG-U133A_2)]. GSE30528 and GSE30529 are the subseries of GSE30122. The glomerular samples of GSE30528 included nine DKD and 13 controls, while the tubulointerstitial samples of GSE30122 included 10 DKD and 12 controls. The diagnosis of DKD patients was based on the presence of diabetes, decreased eGFR (<60 ml/min), increased blood urea nitrogen (BUN), and serum creatinine, as well as proteinuria. Control samples were acquired from nephrectomies, living donors, and healthy people (Woroniecka et al., 2011). To evaluate the quality of GSE30528 and GSE30529, we used the R software to test the intragroup data repeatability *via* the principal component analysis (PCA).

### Identification of differentially expressed genes

Based on the GEOquery package and Limma package of R software, GEO2R enables the users to analyze and visualize the DEGs (Diboun et al., 2006). We used it to obtain significant DEGs between the DKD samples and controls. Based on the platform with annotation information, the probe sets were converted into gene symbols, and the mean value of several probes was analyzed if they were mapped to the same gene. The cutoff values for DEG identification were adjusted *p*-value <0.05 and |logFC (fold change)| >1. The heatmap and volcano plots of glomerular and tubulointerstitial samples were plotted by the ggplot2 package in R software. The Venn diagram was used to identify the common DEGs between GDKD and TDKD.

### Functional enrichment analysis of differentially expressed genes

To understand the functional annotation in the DKD group, we performed the Gene Ontology (GO) and Kyoto Encyclopedia of Genes and Genomes (KEGG) pathway analyses of DEGs. The GO resource is a tool developed to represent the functional knowledge in facilitating computational analysis, and involves three aspects of gene annotations [molecular function (MF), cellular component (CC), and biological process (BP)] (The Gene Ontology Consortium 2019). KEGG analysis was carried out to combine the genomics information and functional analysis by standardizing gene annotations (Kanehisa and Goto 2000). The Benjamini–Hochberg (BH) method was applied to adjust the *p*-value for the false discovery rate (FDR) in our study. The tests were regarded as significant with an adjusted *p*-value threshold of 0.05 and gene count ≥2.

### Protein–protein interaction network generation and subnetwork analysis

The STRING database (http://string-db.org) was used to integrate the DEG-encoded proteins, and their associations with each other to enable comprehensive characterization of the query proteins (Szklarczyk et al., 2017). We used it to create the PPI network by mapping the gene symbols into unique identifiers. The graphical visualization of the generated PPI network was performed by Cytoscape software (Shannon et al., 2003). The molecular complex detection (MCODE) plug-in algorithm of Cytoscape was applied to identify the highly connected subnetworks based on the network connectivity (K-core = 2, degree cutoff = 2, max depth = 100, and node score cutoff = 0.2). Furthermore, the GO function and KEGG pathway analysis of the DEGs included in the subnetworks were analyzed using the clusterProfiler package of R software.

### Identification and analysis of hub genes

We used the cytoHubba algorithm (a plug-in of Cytoscape software) to find the top 20 genes in five algorithms (MCC, MNC, Stress, EPC, and Degree), and regarded the overlapped genes as the hub genes. The interacted network of the hub genes was subsequently created to assign new functions to genes using the GeneMANIA Cytoscape plug-in (Franz et al., 2018).

### Verification of hub gene expression by validation datasets

The mRNA level of the hub genes was validated in the GDKD (GSE47183) and TDKD (GSE47184) dataset. The glomerular samples of GSE47183 included 14 DKD samples and 17 controls, while the tubulointerstitial samples of GSE47184 had 18 DKD samples and six controls. Significance testing was performed using Student’s t-test. A *p*-value <0.05 was considered to be statistically significant.

### Exploration and validation of transcription factors

TRRUST v2 (version 2) (www.grnpedia.org/trrust/) is an online database to investigate the regulation of transcriptional networks. It involves 8,444 TF–target interactions for 800 human TFs, and 6,552 TF–target interactions for 828 mouse TFs (Han et al., 2018). We used the TRRUST v2 tool to acquire the hub gene–related TFs, and constructed the coexpressed network of the TFs and their corresponding hub genes. TFs with adjusted *p*-value <0.05 were identified as statistically significant. Finally, the mRNA expression of the selected TFs was verified in the validation datasets of GDKD and TDKD.

## Results

### Principal component analysis and identification of differentially expressed genes

**FIGURE 1:**
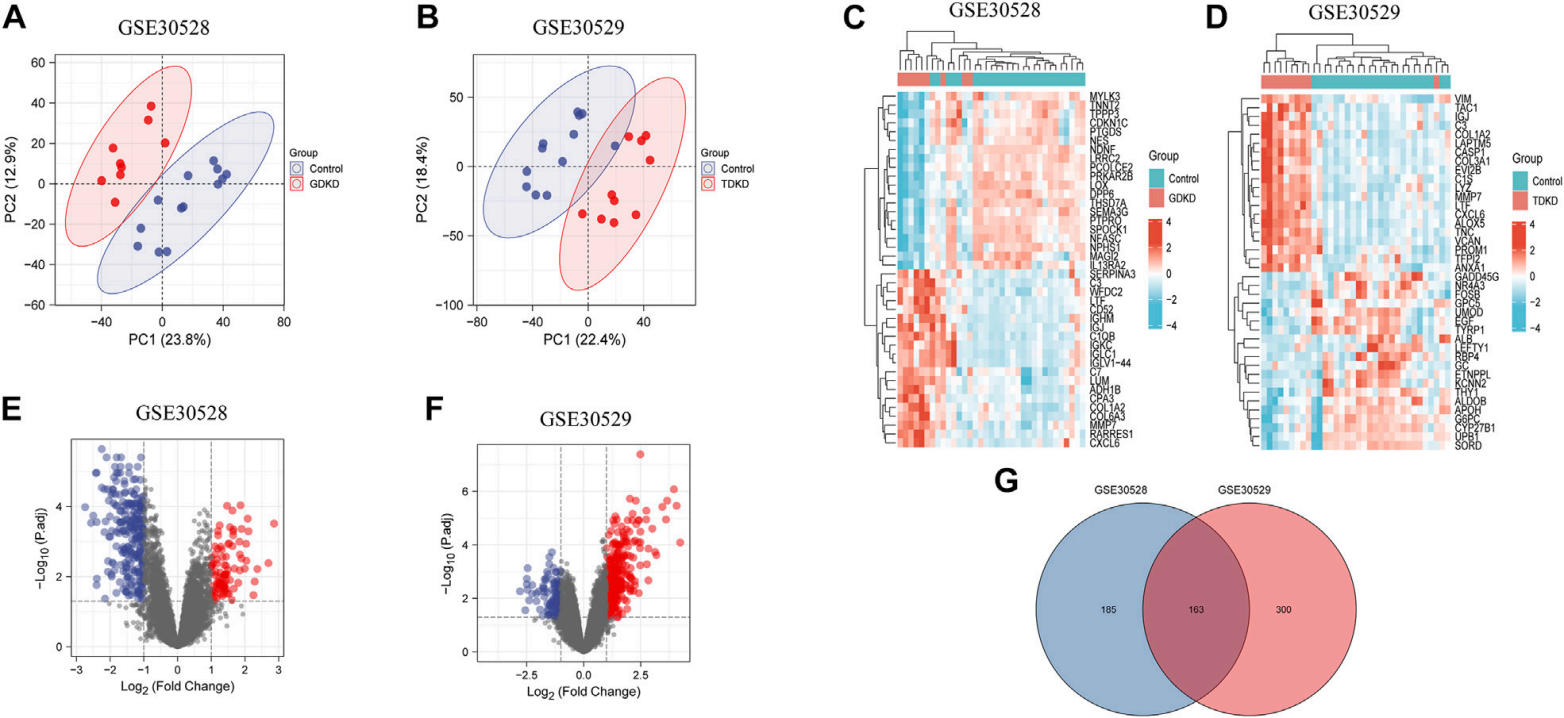
PCA analysis and DEGs identification in GSE30528 and GSE30529. **(A)** PCA of glomerular samples in GDKD (GSE30528). Red dots represent the PCA values of nine glomerular samples in GDKD group, and the blue dots represent the PCA values of 13 glomerular samples in control group. **(B)** PCA of tubulointerstitial samples in TDKD (GSE30529). Red dots represent the PCA values of 10 tubulointerstitial samples in TDKD group, and blue dots represent the PCA values of 12 tubulointerstitial samples in control group. **(C)** Heatmap shows the top 20 significantly upregulated and downregulated DEGs in GSE30528. **(D)** Heatmap shows the top 20 significantly upregulated and downregulated DEGs in GSE30529. **(E)** Volcano diagram of GDKD included 348 DEGs. Red circles represent 93 upregulated DEGs, and blue circles represent 255 downregulated DEGs. **(F)** Volcano diagram of GSE30529 included 463 DEGs. Red circles represent 340 upregulated DEGs, and the blue circles represent 123 downregulated DEGs. **(G)** Venn diagram shows the overlapped DEGs between GDKD and TDKD. GDKD: glomerular diabetic kidney disease; TDKD: tubular diabetic kidney disease; PCA: principal component analysis.

PCA plots showed that the intragroup data repeatability of GDKD and TDKD in GSE30528 and GSE30529 were acceptable. The distances between each human glomerular or tubulointerstitial samples were close in both the control group and the DKD group (Figures 1A,B). In all, 348 DEGs were identified from GDKD, whereas 463 DEGs from TDKD. The heatmap plots of the top 20 DEGs in GDKD and TDKD are displayed in Figures 1C,D, and the volcano plots of DEGs are displayed in Figures 1E,F. Based on the Venn diagram tool, we identified 163 common DEGs between GDKD and TDKD (Figure 1G). After removing the genes with the opposite expression trends in GDKD and TDKD, we finally identified 66 common DEGs (63 were upregulated and three were downregulated) (Table 1).

**TABLE 1:** Details of common DEGs.

Common DEGs	Regulation	Number
LTF, CXCL6, IGJ, MMP7, C3, LAPTM5, TNC, TAC1, CCR2, EVI2A, COL1A2, MS4A4A, IGKC, IGHM, CPA3, GZMA, SERPINA3, CD53, RARRES1, COL6A3, C1QB, CD163, CCL19, CD52, AGR2, CD48, PYCARD, FN1, IL10RA, CCL5, CTSS, TRBC1, MS4A6A, LUM, HS3ST1, THBS2, GZMK, RNASE6, WFDC2, MOXD1, FCER1A, ACKR1, TGFBI, CORO1A, TLR7, IRF8, C1QA, MRC1, FMO3, REG1A, TDO2, LY86, CD2, C7, LTB, DYRK2, GPR18, CD3D, CD1C, LCK, VSIG4, UCP2, and P2RY14.	Upregulated	63	
NELL1, PTPRO, and ETNPPL.	Downregulated	3

### Functional enrichment analysis of common differentially expressed genes

**FIGURE 2:**
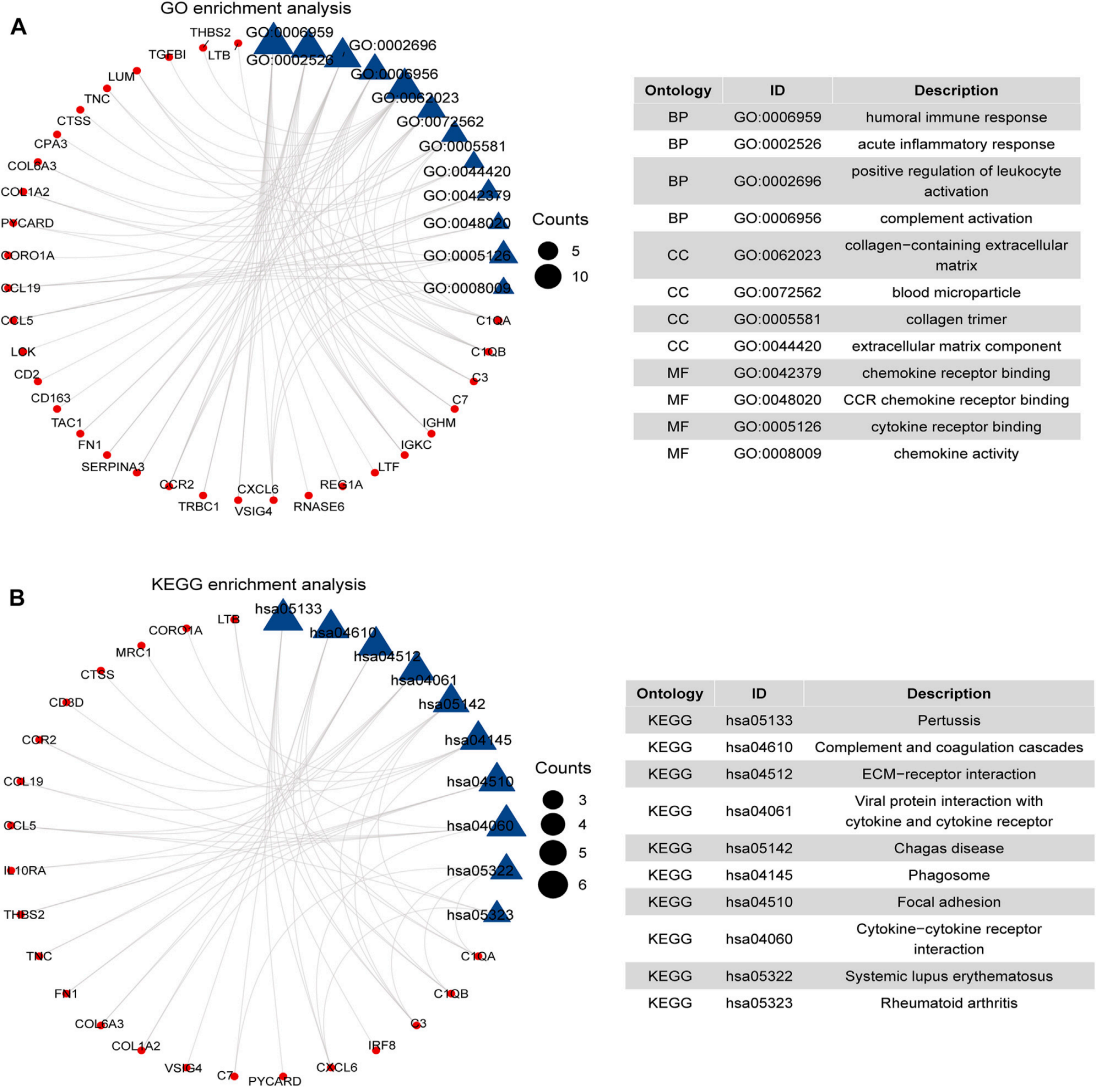
Functional enrichment analysis of 66 DEGs. **(A)** GO enrichment analysis of 66 common DEGs. **(B)** KEGG pathways of 66 common DEGs. We considered adjusted *p*-value < 0.05 as significant. The blue triangle is the term on the right, and the red circle represents the enriched gene of the corresponding pathway.

GO and KEGG pathway analysis were analyzed to evaluate the systematic functional pathway annotations of common DEGs. The top 12 GO-enriched functions are shown in Figure 2A. The common DEGs were enriched in humoral immune response, acute inflammatory response of BPs, collagen-containing extracellular matrix (ECM) of CCs, and chemokine receptor binding and CCR chemokine receptor binding of MFs. According to the KEGG pathways, the common DEGs were enriched in complement and coagulation cascades, ECM–receptor interaction, focal adhesion, and viral protein interaction with cytokine and cytokine receptors (Figure 2B). These results suggest that the inflammatory-related pathways and ECM, especially chemokines and cytokines, are associated with the development and progression of GDKD and TDKD.

### Protein–protein interaction network generation and subnetwork analysis

**FIGURE 3:**
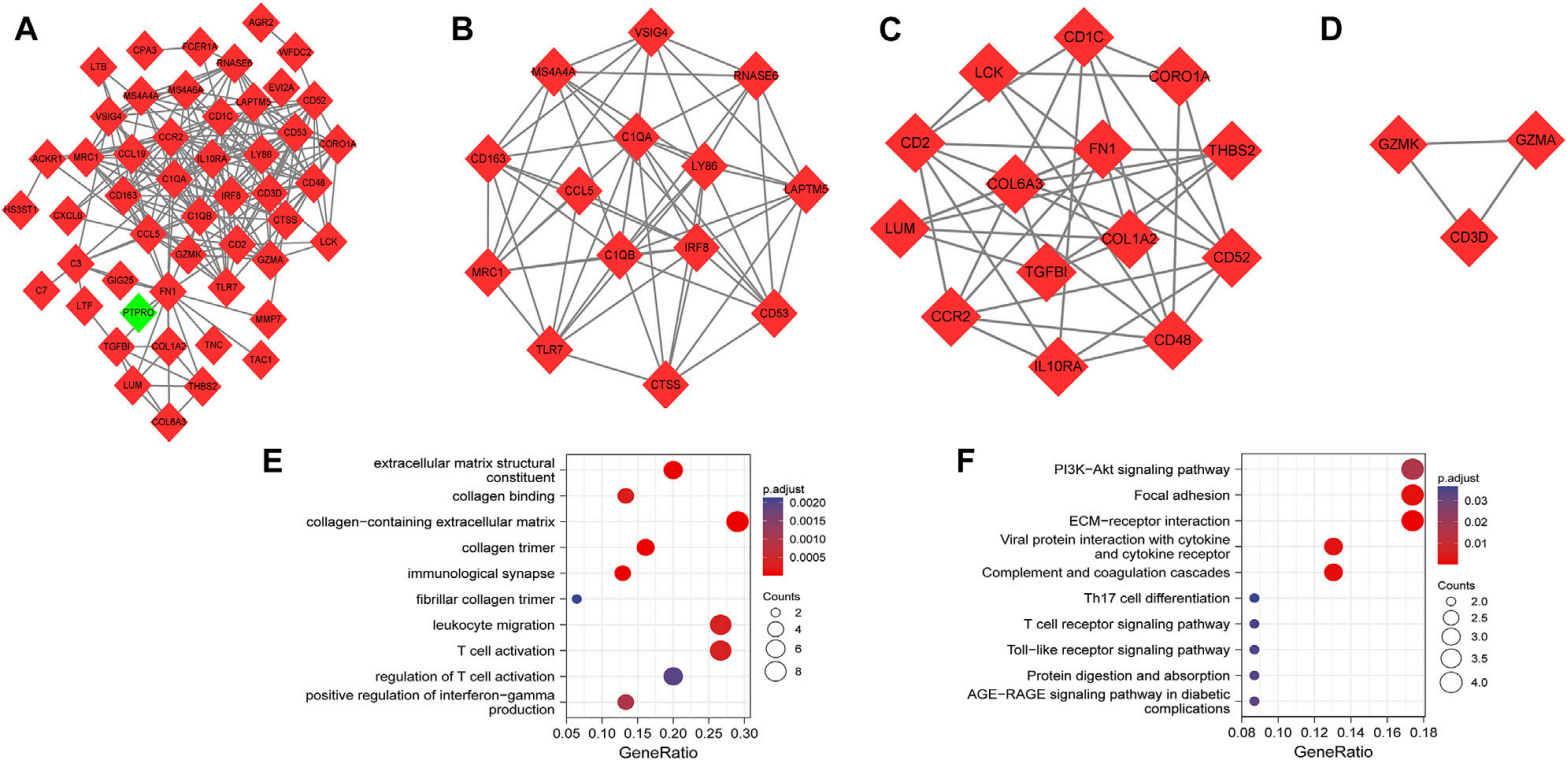
PPI network, highly connected subnetworks, and enrichment analyses of genes involved in subnetworks. **(A)** The PPI network included 50 nodes and 217 edges. Red diamonds represent upregulated genes, while green diamond represents downregulated genes. **(B–D)** Three highly connected subnetworks have the gene-level score of 9.077, 6, and 3, respectively. Red diamonds represent upregulated genes. **(E)** GO analysis of the total genes involved in subnetworks. **(F)** KEGG pathways of total genes involved in subnetworks. Size of the circles represents the number of enriched genes.

We generated the PPI network based on a combined score >0.4. As illustrated in Figure 3A, the PPI network consisted of 50 nodes and 217 edges. Three highly connected subnetworks were identified by the MCODE plug-in algorithm. These involved 31 nodes and 101 edges with a large gene-level score of 9.077, 6, and 3, respectively (Figures 3B–D). Based on GO functional analysis, the genes in the subnetworks were primarily enriched in ECM and inflammatory responses mediated by cytokines and chemokines (Figure 3E). Based on the KEGG pathway analysis, the genes in the subnetworks were mainly enriched in ECM–receptor interaction, complement and coagulation cascades, focal adhesion, and viral protein interaction with cytokine and cytokine receptors (Figure 3F).

### Identification and analysis of hub genes

**FIGURE 4:**
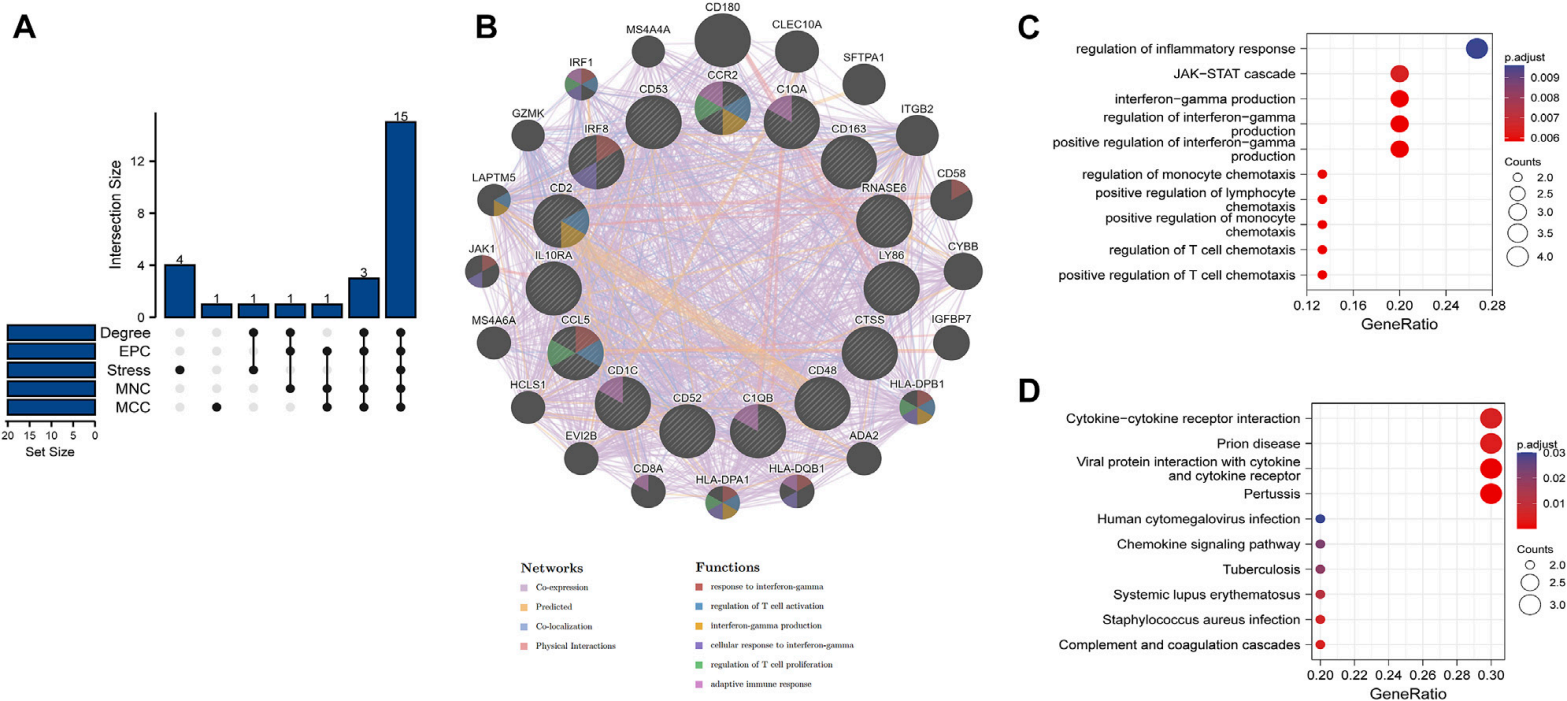
Venn diagram, coexpressed network of hub genes, and enrichment analyses of hub genes **(A)** 15 hub genes were identified by five algorithms. **(B)** Coexpressed network of hub genes was shown with the GeneMANIA online tool. The hub genes were located in the inner circle, while the functionally similar genes were located in the outer circle. **(C)** GO enrichment analysis of hub genes was constructed using a bubble plot. **(D)** KEGG pathway of hub genes was constructed using a bubble plot. Adjusted *p*-value < 0.05 was considered as significant.

Based on the overlapped parameters of the top 20 genes in the five algorithms, 15 hub genes, namely, IL10RA, IRF8, LY86, C1QA, C1QB, CD53, CD1C, CTSS, CCR2, CD163, CCL5, CD48, RNASE6, CD52, and CD2 were identified using the Venn diagram (Figure 4A). A detailed description of the hub genes is provided in Table 2. We uploaded the hub genes on the GeneMANIA tool to explore functionally similar genes. Among them, the coexpressed network showed the functional annotation with coexpressed for 63.56%, predicted for 20.09%, colocalized for 10.08%, and physical interaction for 6.27% (Figure 4B) Their functions were mainly enriched in cellular response to interferon-gamma (IFN-γ), IFN-γ production, regulation of T-cell activation, and regulation of T-cell proliferation. This emphasizes the critical role of cytokines and chemokines in DKD. GO analysis of the hub genes showed that the JAK-STAT cascade, regulation of IFN-γ production, positive regulation of T-cell chemotaxis, and regulation of T-cell chemotaxis were the significantly enriched functions in DKD (Figure 4C). KEGG pathway analysis showed that cytokine–cytokine receptor interaction, complement and coagulation cascades, and pertussis were the significantly enriched pathways in DKD (Figure 4D).

**TABLE 2:** Detailed information of hub genes.

Gene symbol	Full name	Function
IL10RA	Interleukin 10 receptor subunit alpha	IL10RA is a receptor for interleukin 10 that has been shown to mediate the immunosuppressive signal of interleukin 10, and thus inhibits the synthesis of proinflammatory cytokines
IRF8	Interferon regulatory factor 8	It is a transcription factor of the interferon regulatory factor family and is associated with interferon-gamma signaling and cytokine signaling.
LY86	Lymphocyte antigen 86	It acts upstream of or within the positive regulation of lipopolysaccharide-mediated signaling pathway.
C1QA	Complement C1q subcomponent subunit A	It encodes the A-chain polypeptide of serum complement subcomponent C1q, and participates in and is associated with lupus erythematosus and glomerulonephritis.
C1QB	Complement C1q subcomponent subunit B	It encodes the B-chain polypeptide of serum complement subcomponent C1q and participates in immune response lectin-induced complement pathway and innate immune system.
CD53	CD53 molecule	It plays a significant role in the regulation of cell development, activation, growth, and motility.
CD1C	CD1c molecule	CD1C is an antigen-presenting protein that binds self and nonself-lipid and glycolipid antigens and presents them to T-cell receptors on natural killer T-cells.
CTSS	Cathepsin S	CTSS usually remodel components of the extracellular matrix and implicated in many inflammatory and autoimmune diseases
CCR2	Chemokine C-C motif receptor 2	CCR2 is a receptor of CCL2 and regulates monocyte infiltration in inflammatory diseases
CD163	CD163 molecule	It may function as an innate immune sensor for bacteria and inducer of local inflammation
CCL5	C-C motif chemokine ligand 5	CCL5 is a chemokine, a member of the CC subfamily, which functions as a chemoattractant for blood monocytes, memory T helper cells, and eosinophils.
CD48	CD48 molecule	CD48 is a ligand for CD2 and might facilitate interaction between activated lymphocytes.
RNASE6	Ribonuclease A family member k6	It is a member of the ribonuclease A superfamily and involved in defensins and innate immune system.
CD52	CD52 molecule	CD52 may play a role in carrying and orienting carbohydrate.
CD2	CD2 molecule	It is a surface antigen found on all peripheral blood T-cells and mediates adhesion between T-cells.

### Verification of hub gene expression in validation datasets

**FIGURE 5:**
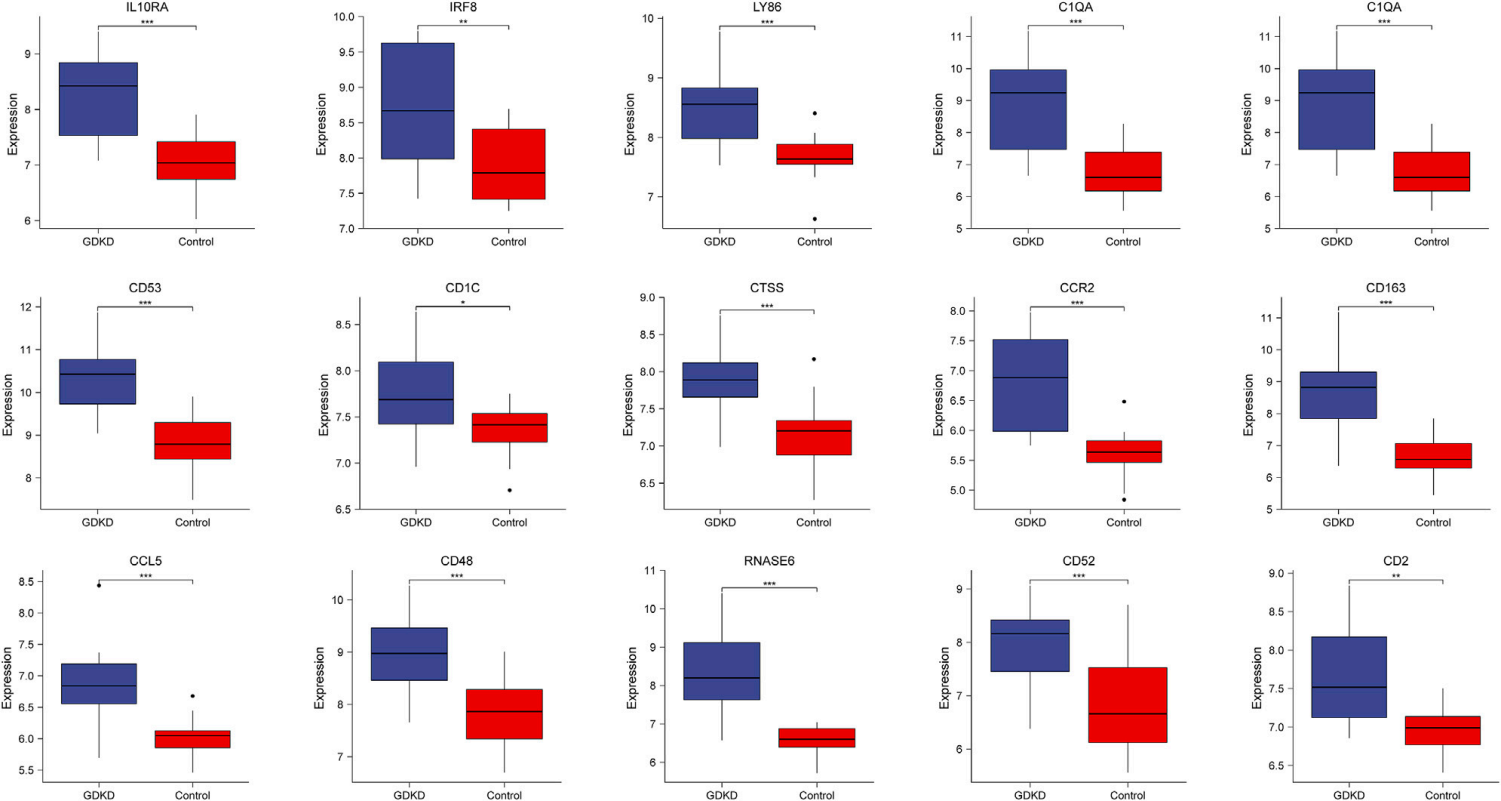
mRNA level of hub genes in GSE47183. All hub genes are upregulated in GDKD samples compared with controls. *p < 0.05, *p < 0.01, ***p < 0.001. GDKD: glomerular diabetic kidney disease.

**FIGURE 6:**
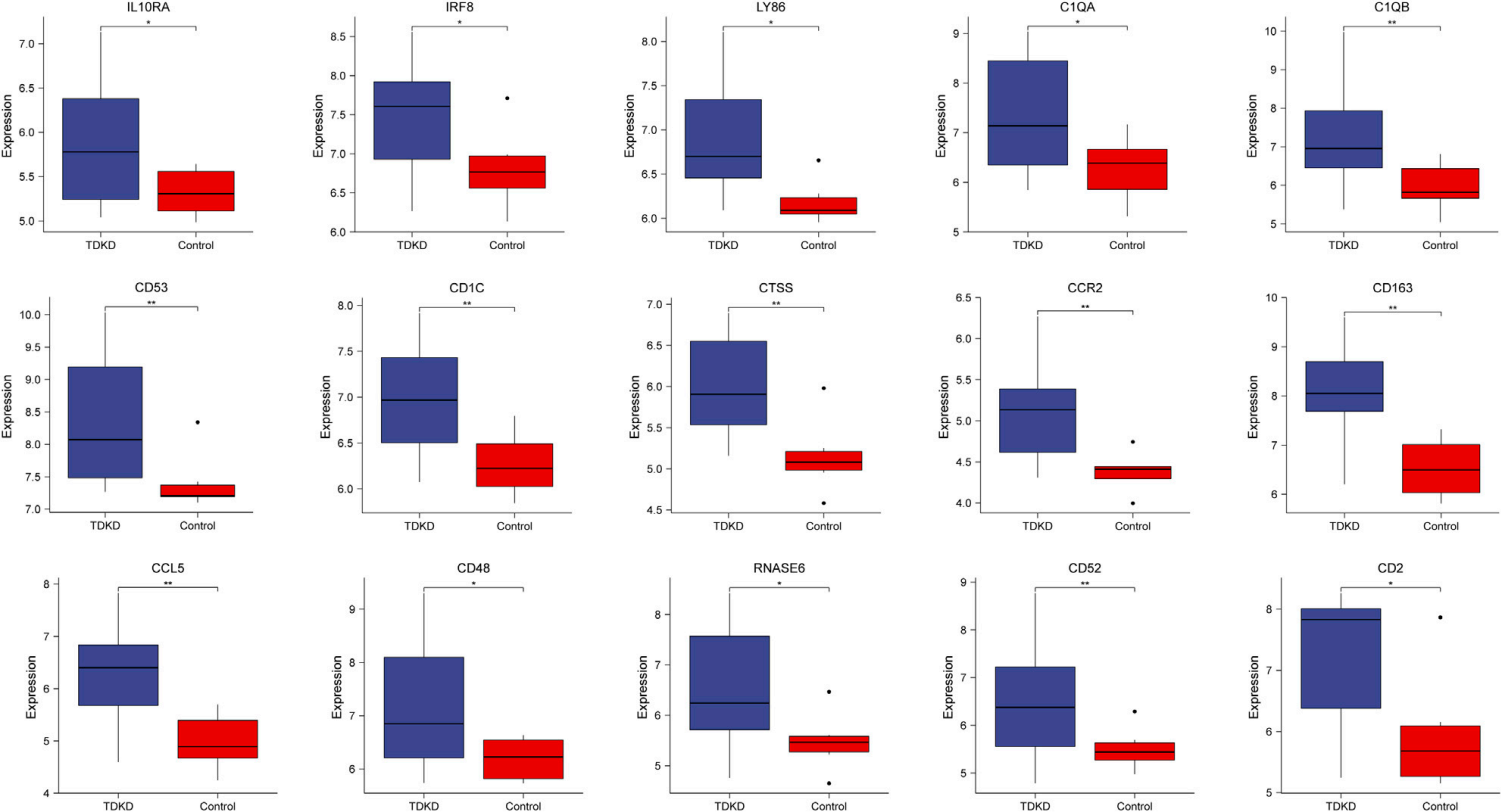
mRNA level of hub genes in GSE47I84. All hub genes are upregulated in TDKD samples compared with controls. *p < 0.05. **p < 0.01, ***p < 0.001. TDKD: tubular diabetic kidney disease.

To verify the reliability of the identified hub genes, we chose two independent expression profiles containing GDKD and TDKD, and explored the mRNA expression level of hub genes. We identified that 15 hub genes were upregulated in GDKD (Figure 5). The upregulated expression trend of 15 hub genes was also observed in TDKD (Figure 6).

### Exploration and validation of transcription factors

**FIGURE 7:**
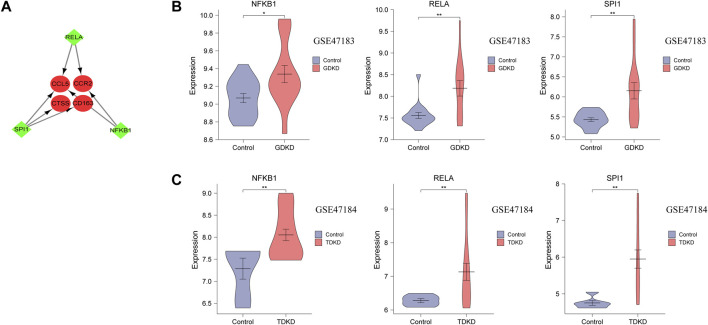
TFs regulatory network and TFs expression in GSE47183 and GSE47184. **(A)** Green diamonds represent TFs, and red circles represent hub genes. **(B,C)** mRNA level of TFs in GSE47183 and GSE47184. *p < 0.05, **p < 0.01, ***p < 0.001. GDKD: glomerular diabetic kidney disease, TDKD: tubular diabetic kidney disease.

Three TFs (SPI1, RELA, and NFKB1) that have regulatory relationships with the hub genes were obtained using the TRRUST database (Figure 7A and Table 3). In addition, the mRNA expression of these three TFs showed significantly increased levels in GDKD and TDKD (Figures 7B,C). These TFs were coordinately involved in regulating the CCR2, CCL5, CD163, and CTSS hub genes.

**TABLE 3:** Key transcription factors (TFs) of hub genes.

Key TFs	Description	Q value	List of overlapped genes
SPI1	Spleen focus forming virus (SFFV) proviral integration oncogene spi1	5.98E-05	CCL5, CTSS, CD163
RELA	V-rel reticuloendotheliosis viral oncogene homolog A (avian)	0.0313	CCR2, CCL5
NFKB1	Nuclear factor of kappa light polypeptide gene enhancer in B-cells 1	0.0313	CCL5, CCR2

## Discussion

DKD is a clinically and pathophysiologically heterogeneous disease, and its etiology is not completely understood. DKD is generally classified as a noninflammatory disease. However, research during these years has established a clear link of glomerular and tubulointerstitial lesions of DKD with the systematic or local inflammatory responses (Flyvbjerg 2017). This suggests that the inflammatory response is a key mechanism of DKD. Inflammatory mediators, including circulating tumor necrosis factor alpha (TNF-α), TNF receptor 1(TNFR1), and serum amyloid A, are emerging as potential biomarkers and therapeutic targets for DKD (Alicic et al., 2018). Nonetheless, relevant results from trials of multiple biomarkers are not consistent. Hence, pathways associated with the pathogenesis of DKD must be elucidated to develop novel biomarkers.

As an important driver of inflammatory responses, activated complement strengthens the capacity of antibodies and phagocytic cells to clear the pathogenic microorganisms and damaged cells. Current studies have revealed that the complement system is significantly involved in the development of glomerular and tubulointerstitial lesions in DKD (Flyvbjerg 2017). Increased expression of proinflammatory cytokines produced by damaged glomerular and tubular cells is of high significance in DKD. These proinflammatory cytokines include IL-1, TNF, and CCL5/RANTES (Navarro-González et al., 2011). In DKD, the activated endothelium under high glucose and abnormal hemodynamic conditions upregulate the expression of cytokines and chemokines, and results in renal impairment (Langer and Chavakis 2009; Moreno et al., 2014). The inflammatory response is thought to contribute to renal damage of DKD through two mechanisms. One dominant mechanism is the direct interaction between the glomerular and tubular cells. The other is *via* the release of chemokines, cytokines, and profibrotic factors. Once activated by these released mediators, the renal stromal cells release large numbers of chemokines, which subsequently facilitate the infiltration of leukocytes. These events amplify the inflammatory process, leading to further damage to renal structure and function (Luis-Rodríguez et al., 2012; Pichler et al., 2017).

In the present work, we reanalyzed GSE30528 and GSE30529 and identified 66 common DEGs in both the human glomerular and tubulointerstitial samples of DKD. Among them, 15 DEGs, including IL10RA, IRF8, LY86, C1QA, C1QB, CD53, CD1C, CTSS, CCR2, CD163, CCL5, CD48, RNASE6, CD52, and CD2, were identified as the hub genes. Functional enrichment analyses indicated that not only inflammatory-related signaling pathways but also ECM-related pathways were enriched in glomerular and tubulointerstitial tissues of DKD, which had not been extensively studied in the original article (Woroniecka et al., 2011). In particular, chemokines and cytokines, such as regulation of IFN-γ production, regulation of monocyte chemotaxis, positive regulation of lymphocyte chemotaxis, regulation of T-cell chemotaxis, and cytokine–cytokine receptor interaction, are jointly involved in the glomerular and tubulointerstitial lesions of DKD, further highlighting the importance of inflammatory response in the onset and pathogenesis of DKD. Moreover, deposition of ECM proteins (including collagen, laminin, and fibronectin) in the renal glomerulus and tubulointerstitial can lead to glomerulosclerosis and tubulointerstitial fibrosis, which, in turn, constitute the characteristic lesions of DKD (Hu et al., 2015). The JAK-STAT cascade is also enriched in DKD, and this enrichment was particularly relevant to the pathogenesis of DKD. High glucose activates the JAK-STAT pathway in mesangial cells, with concomitant mesangial cell proliferation and accumulation of ECM proteins (Wang et al., 2002). Potential roles of the JAK/STAT pathway in the human glomerular and tubulointerstitial tissues of DKD have also been validated *via* transcriptomic approaches (Marrero et al., 2006). Compared with the original article, we also found that three TFs (SPI1, RELA, and NFKB1) might have a regulatory relationship with specific hub genes. By verifying their expression levels in validation datasets, we found that three TFs were upregulated in human glomerular and tubulointerstitial samples of DKD. These TFs coordinately regulate hub gene expression (CCR2, CCL5, CD163, and CTSS).

Hyperglycemia, intraglomerular hypertension, and overactivated neurohormone promote the activation of inflammatory and profibrotic biological processes, thus resulting in glomerular and tubulointerstitial lesions in DKD. Under such conditions, anti-inflammatory and antifibrosis effects may facilitate the use of renal protective agents, such as adhesion molecule inhibitors, sodium-dependent glucose transporters 2 inhibitors, and IL-1β antagonists in combination with the conventional renin-angiotensin-aldosterone system (RAAS) inhibitors in patients with DKD (Lytvyn et al., 2020; Moisi et al., 2020; Moisi et al., 2021). It should be noted that the observed transcriptional alternations may be the drivers of the renal structural and functional changes in DKD. Thus, identifying the biomarkers that indicate the potential pathogenesis behind the development and progression of DKD may contribute to developing more targeted therapies in personalized patients (Barrera-Chimal and Jaisser 2020).

Infiltration of immune cells in renal tissues is a defining feature of early DKD, and is associated with the increased risk of progression in DKD. Chemokines and their receptors are the chief molecules of immune cells that are involved in inflammatory responses (Pérez-Morales et al., 2019). C–C motif chemokine ligand 2 (CCL2), C–C motif chemokine ligand 5 (CCL5), and their receptors CCR2 and CCR5, respectively, are reported to have a close connection with DKD. As the major receptor of CCL2, CCR2 is mainly characterized by the distribution of macrophages and monocytes in renal tissues (Navarro-González et al., 2011). It mediates interstitial inflammation, tubulointerstitial fibrosis, podocyte damage, and glomerulosclerosis (Chow et al., 2006; Park et al., 2008; Lee et al., 2009). RO5234444, an inhibitor of CCR2, can reduce the infiltration of inflammatory cells in diabetic mice and block the progression of DKD. In human tubulointerstitial samples of DKD, the expression of CCL2 was increased (Satirapoj 2018), and urinary excretion of CCL2 contributed to renal tubular damage and albuminuria (Morii et al., 2003). Previous studies suggested that the CCL2/CCR2 signaling pathway induces podocyte injury and increases podocyte permeability to albumin (Morii et al., 2003). However, treatment with a CCR2 antagonist (RS102895) attenuated the morphological changes including the thickening of glomerular filtration membrane in diabetic mice, expansion of the mesangium, and the effacement of podocyte foot processes (Seok et al., 2013). CCL5 is expressed by multiple cell types, namely, fibroblasts, mesangial cells, and renal tubular epithelial cells. It actively participates in the recruitment of monocytes, macrophages, and T-cells to the renal glomerular and tubulointerstitial (Wolf et al., 1993; Appay and Rowland-Jones 2001). Increased expression of CCL5 in mesangial cells and tubular cells is apparently induced by the NF-κB–dependent pathways (Schmid et al., 2006), enhanced glomerular filtration of growth factors, and other abnormal factors related to DKD (Wolf et al., 1997). In patients with type 2 diabetes mellitus and overt nephropathy, the renal biopsies of CCL5 are significantly elevated, especially in tubular cells, and this increase is directly correlated with proteinuria and interstitial cellular infiltration (Mezzano et al., 2004). Evidence of association between Japanese patients and DKD indicated that the CCL5 promoter-28G genotype and CCR5 promoter-59029A genotype might additively be associated with DKD. This further emphasizes the key role of the CCL5/CCR5 signaling pathway in the development of DKD (Nakajima et al., 2003).

One notable feature of DKD is the influx of inflammatory cells in the renal tissues. This has been reported to have a connection with the differences in macrophage phenotype and function, including the proinflammatory M1 phenotype and anti-inflammatory M2 phenotype. CD163, a specific marker of the anti-inflammatory monocyte and M2 macrophages, is capable of transmitting signals upon and thus releasing the anti-inflammatory mediators such as IL-10 (Ricardo et al., 2008; Van Gorp et al., 2010). To our knowledge, macrophages isolated from diabetic kidneys indicate that both M1 and M2 phenotypes coexist ( You et al., 2013). However, the expression of anti-inflammatory M2 phenotype (CD163) is lower than that of proinflammatory M1 phenotype (Ricardo et al., 2008; You et al., 2013). A histological study of renal autopsy samples from individuals with diabetes mellitus revealed a 2:1 ratio of M1:M2 cells in the glomerulus and tubulointerstitium. Moreover, it found that the accumulation of M2 cells was associated with interstitial fibrosis and tubular atrophy (Klessens et al., 2017). The observed imbalance between M1 and M2 (CD163) phenotype might drive inflammation and fibrosis in the pathogenesis of DKD. Thus, targeting CD163 may be a promising approach for the inflammatory-based treatment of DKD.

Early endothelial dysfunction is thought to be important for the pathogenesis of DKD (Nakagawa et al., 2011). The traditional view holds that the loss of endothelial nitric oxide synthase is a cause of endothelial dysfunction. However, a recent study showed that macrophage-derived cathepsin S (CTSS) was likely to accelerate endothelial damage in DKD (Kumar Vr et al., 2016). Cathepsin S, a member of the family of cysteine proteases, is associated with protein degradation in the endosomal/lysosomal pathway, and cleaves substrates like protease-activated receptor-2 (PAR-2) (Wilkinson et al., 2015). Increased levels of CTSS in serum have been reported in many diseases, and relate to diabetes mellitus and heart disease (Liu et al., 2006; Vesey et al., 2007). Macrophage-derived CTSS mediates cell damage by activating PAR-2 located on endothelial cell surface. The application of the PAR-2 inhibitor (GB83) reduced endothelial cell injury and glomerulosclerosis, and this provided the first evidence for the pathogenic role of PAR-2 in DKD (Nikolic-Paterson, 2016). More importantly, treatment of diabetic kidney disease in uninephrectomized db/db mice with a selective CTSS inhibitor (RO5461111) improved endothelial injury, albuminuria, and glomerulosclerosis as well as albumin leakage in the retina (Kumar Vr et al., 2016). The observed benefits were correlated with renoprotection against podocyte injury and loss, endothelial cell injury and loss, and decrease of macrophage infiltration and inflammatory biomarkers (Kumar Vr et al., 2016).

A previous study examined the inflammatory and profibrotic genes associated with DKD. It found that the expression of CCR2, MOXD1, COL6A3, COL1A2, PYCARD, and C7 was increased in human kidney biopsy of DKD, as well as in DKD mice *in vivo* (Chen et al., 2022). Our article also found that novel genes related to inflammatory responses like IL10RA, IRF8, LY86, CD53, CD48, RNASE6, and CD52 are highly expressed in both glomerular and tubulointerstitial samples of DKD. Indeed, the inflammatory-related signaling pathway, which was noticed to involve a large number of genes, would mediate the activation of cytokines and chemokines.

Using the integrated bioinformatics analysis, Jiao et al. (2021) showed that C1q and C3 are the dominant complement-related mediators in GDKD. They are upregulated in the glomerular biopsy samples of DKD, and patients with C1q and C3 deposition had more severe glomerular class. In addition, Xu et al. (2021) showed that immune response played a significant role in TDKD, and VCAN was identified as a crucial gene in the immune processes during TDKD progression. However, few articles focused on the shared genes and biological pathways between the glomerular and tubulointerstitial lesions in DKD. Since renal glomerular and tubulointerstitial damages both have crucial roles in the progression of DKD, we pay more attention to the common genes and TFs between GDKD and TDKD. This microarray data analysis has been proven to be successful in other diseases (Fang et al., 2020; Su et al., 2021). We further explored the associated TFs, and validated their expression levels in validation datasets of TDKD and GDKD. We believe that our study will offer additional insights into the biological pathways and markers to enable the prevention of DKD progression.

## Limitations

There are several limitations to existing studies. 1) Most transcription studies of DKD were carried out for European populations and paid relatively less attention to non-European populations with a high incidence of DKD. Therefore, the summarized results might lack global representation. 2) The current research merely analyzed the microarray datasets of DKD at the transcription level, without the involvement of genomics, proteomics, and metabolomics. This may lead to an incomplete understanding of the mechanisms and biomarkers of DKD. 3) The microarray datasets are unable to discover gene profiles with the underdeveloped and less known sites, and are restricted to a limited number of gene sites owing to their low sensitivity. In summary, the potential directions for future research should take these factors into account.

## Conclusion

This study used the microarray data analysis to analyze and compare the common DEGs, biological pathways, hub genes, and TFs between the glomerular and tubulointerstitial lesions in DKD. This will enable physicians to understand the molecular pathomechanisms of DKD and offer an early and precise diagnosis for patients. Future practices are required to focus on clinical applications, with sustained efforts to increase awareness about the importance of molecular targets for treating DKD.

## Data Availability

The datasets presented in this study can be found in online repositories. The names of the repository/repositories and accession number(s) can be found in the article/Supplementary Material.

## References

[B1] Alicic R. Z. Johnson E. J. Tuttle K. R. (2018). Inflammatory mechanisms as new biomarkers and therapeutic targets for diabetic kidney disease. Adv. Chronic Kidney Dis. 25, 181–191. 10.1053/j.ackd.2017.12.002 29580582

[B2] Alicic R. Z. Rooney M. T. Tuttle K. R. (2017). Diabetic kidney disease: Challenges, progress, and possibilities. Clin. J. Am. Soc. Nephrol. 12, 2032–2045. 10.2215/CJN.11491116 28522654 PMC5718284

[B3] Anders H. J. Huber T. B. Isermann B. Schiffer M. (2018). CKD in diabetes: Diabetic kidney disease versus nondiabetic kidney disease. Nat. Rev. Nephrol. 14, 361–377. 10.1038/s41581-018-0001-y 29654297

[B4] Appay V. Rowland-Jones S. L. (2001). RANTES: A versatile and controversial chemokine. Trends Immunol. 22, 83–87. 10.1016/s1471-4906(00)01812-3 11286708

[B5] Arora M. K. Singh U. K. (2013). Molecular mechanisms in the pathogenesis of diabetic nephropathy: An update. Vasc. Pharmacol. 58, 259–271. 10.1016/j.vph.2013.01.001 23313806

[B6] Barrera-Chimal J. Jaisser F. (2020). Pathophysiologic mechanisms in diabetic kidney disease: A focus on current and future therapeutic targets. Diabetes Obes. Metab. 22, 16–31. 10.1111/dom.13969 32267077

[B7] Barrett T. Wilhite S. E. Ledoux P. Evangelista C. Kim I. F. Tomashevsky M. (2013). NCBI GEO: Archive for functional genomics data sets--update. Nucleic Acids Res. 41, D991–D995. 10.1093/nar/gks1193 23193258 PMC3531084

[B8] Bell S. Fletcher E. H. Brady I. Looker H. C. Levin D. Joss N. (2015). End-stage renal disease and survival in people with diabetes: A national database linkage study. QJM 108, 127–134. 10.1093/qjmed/hcu170 25140030 PMC4309927

[B9] Budge K. Dellepiane S. Yu S. M. Cravedi P. (2021). Complement, a therapeutic target in diabetic kidney disease. Front. Med. 7, 599236. 10.3389/fmed.2020.599236 PMC7858668 33553201

[B10] Chen J. Luo S. F. Yuan X. Wang M. Yu H. J. Zhang Z. (2022). Diabetic kidney disease-predisposing proinflammatory and profibrotic genes identified by weighted gene co-expression network analysis (WGCNA). J. Cell. Biochem. 123, 481–492. 10.1002/jcb.30195 34908186

[B11] Chobot A. Górowska-Kowolik K. Sokołowska M. Jarosz-Chobot P. (2018). Obesity and diabetes-Not only a simple link between two epidemics. Diabetes. Metab. Res. Rev. 34, e3042. 10.1002/dmrr.3042 29931823 PMC6220876

[B12] Chow F. Y. Nikolic-Paterson D. J. Ozols E. Atkins R. C. Rollin B. J. Tesch G. H. (2006). Monocyte chemoattractant protein-1 promotes the development of diabetic renal injury in streptozotocin-treated mice. Kidney Int. 69, 73–80. 10.1038/sj.ki.5000014 16374426

[B13] Collins A. J. Foley R. N. Gilbertson D. T. Chen S. C. (2015). United States renal data system public health surveillance of chronic kidney disease and end-stage renal disease. Kidney Int. Suppl. 2011 (5), 2–7. 10.1038/kisup.2015.2 PMC4455192 26097778

[B14] Diboun I. Wernisch L. Orengo C. A. Koltzenburg M. (2006). Microarray analysis after RNA amplification can detect pronounced differences in gene expression using limma. BMC Genomics 7, 252. 10.1186/1471-2164-7-252 17029630 PMC1618401

[B15] Duni A. Liakopoulos V. Roumeliotis S. Peschos D. Dounousi E. (2019). Oxidative stress in the pathogenesis and evolution of chronic kidney disease: Untangling ariadne's thread. Int. J. Mol. Sci. 20, 3711. 10.3390/ijms20153711 PMC6695865 31362427

[B16] Fang X. Duan S. F. Gong Y. Z. Wang F. Chen X. L. (2020). Identification of key genes associated with changes in the host response to severe burn shock: A bioinformatics analysis with data from the gene expression Omnibus (GEO) database. J. Inflamm. Res. 13, 1029–1041. 10.2147/JIR.S282722 33293847 PMC7718973

[B17] Flyvbjerg A. (2017). The role of the complement system in diabetic nephropathy. Nat. Rev. Nephrol. 13, 311–318. 10.1038/nrneph.2017.31 28262777

[B18] Franz M. Rodriguez H. Lopes C. Zuberi K. Montojo J. Bader G. D. (2018). GeneMANIA update 2018. Nucleic Acids Res. 46, W60–W64. 10.1093/nar/gky311 29912392 PMC6030815

[B19] Han H. Cho J. W. Lee S. Yun A. Kim H. Bae D. (2018). TRRUST v2: An expanded reference database of human and mouse transcriptional regulatory interactions. Nucleic Acids Res. 46, D380–D386. 10.1093/nar/gkx1013 29087512 PMC5753191

[B20] Hu C. Sun L. Xiao L. Han Y. Fu X. Xiong X. (2015). Insights into the mechanisms involved in the expression and regulation of extracellular matrix proteins in diabetic nephropathy. Curr. Med. Chem. 22, 2858–2870. 10.2174/0929867322666150625095407 26119175 PMC4863711

[B21] Jiao Y. Jiang S. Wang Y. Yu T. Zou G. Zhuo L. (2021). Activation of complement C1q and C3 in glomeruli might accelerate the progression of diabetic nephropathy: Evidence from transcriptomic data and renal histopathology. J. Diabetes Investig. 13, 839–849. 10.1111/jdi.13739 PMC9077730 34932275

[B22] Kanehisa M. Goto S. (2000). KEGG: Kyoto encyclopedia of genes and genomes. Nucleic Acids Res. 28, 27–30. 10.1093/nar/28.1.27 10592173 PMC102409

[B23] Klessens C. Zandbergen M. Wolterbeek R. Bruijn J. A. Rabelink T. J. Bajema I. M. (2017). Macrophages in diabetic nephropathy in patients with type 2 diabetes. Nephrol. Dial. Transpl. 32, 1322–1329. 10.1093/ndt/gfw260 27416772

[B24] Kumar Vr S. Darisipudi M. N. Steiger S. Devarapu S. K. Tato M. Kukarni O. P. (2016). Cathepsin S cleavage of protease-activated receptor-2 on endothelial cells promotes microvascular diabetes complications. J. Am. Soc. Nephrol. 27, 1635–1649. 10.1681/ASN.2015020208 26567242 PMC4884104

[B25] Langer H. F. Chavakis T. (2009). Leukocyte-endothelial interactions in inflammation. J. Cell. Mol. Med. 13, 1211–1220. 10.1111/j.1582-4934.2009.00811.x 19538472 PMC2861890

[B26] Lee E. Y. Chung C. H. Khoury C. C. Yeo T. K. Pyagay P. E. Wang A. (2009). The monocyte chemoattractant protein-1/CCR2 loop, inducible by TGF-beta, increases podocyte motility and albumin permeability. Am. J. Physiol. Ren. Physiol. 297, F85–F94. 10.1152/ajprenal.90642.2008 PMC2711714 19420107

[B27] Liu J. Ma L. Yang J. Ren A. Sun Z. Yan G. (2006). Increased serum cathepsin S in patients with atherosclerosis and diabetes. Atherosclerosis 186, 411–419. 10.1016/j.atherosclerosis.2005.08.001 16140306

[B28] Liyanage T. Ninomiya T. Jha V. Neal B. Patrice H. M. Okpechi I. (2015). Worldwide access to treatment for end-stage kidney disease: A systematic review. Lancet 385, 1975–1982. 10.1016/S0140-6736(14)61601-9 25777665

[B29] López-Novoa J. M. Rodríguez-Peña A. B. Ortiz A. Martínez-Salgado C. López Hernández F. J. (2011). Etiopathology of chronic tubular, glomerular and renovascular nephropathies: Clinical implications. J. Transl. Med. 9, 13. 10.1186/1479-5876-9-13 21251296 PMC3034700

[B30] Lozano R. Naghavi M. Foreman K. Lim S. Shibuya K. Aboyans V. (2012). Global and regional mortality from 235 causes of death for 20 age groups in 1990 and 2010: A systematic analysis for the global burden of disease study 2010. Lancet 380, 2095–2128. 10.1016/S0140-6736(12)61728-0 23245604 PMC10790329

[B31] Luis-Rodríguez D. Martínez-Castelao A. Górriz J. L. De-Álvaro F. Navarro-González J. F. (2012). Pathophysiological role and therapeutic implications of inflammation in diabetic nephropathy. World J. Diabetes 3, 7–18. 10.4239/wjd.v3.i1.7 22253941 PMC3258536

[B32] Lytvyn Y. Bjornstad P. van Raalte D. H. Heerspink H. L. Cherney D. (2020). The new biology of diabetic kidney disease-mechanisms and therapeutic implications. Endocr. Rev. 41, bnz010. 10.1210/endrev/bnz010 31633153 PMC7156849

[B33] Marrero M. B. Banes-Berceli A. K. Stern D. M. Eaton D. C. (2006). Role of the JAK/STAT signaling pathway in diabetic nephropathy. Am. J. Physiol. Ren. Physiol. 290, F762–F768. 10.1152/ajprenal.00181.2005 16527921

[B34] Mezzano S. Aros C. Droguett A. Burgos M. E. Ardiles L. Flores C. (2004). NF-kappaB activation and overexpression of regulated genes in human diabetic nephropathy. Nephrol. Dial. Transpl. 19, 2505–2512. 10.1093/ndt/gfh207 15280531

[B35] Moisi M. I. Bungau S. G. Vesa C. M. Diaconu C. C. Behl T. Stoicescu M. (2021). Framing cause-effect relationship of acute coronary syndrome in patients with chronic kidney disease. Diagn. (Basel) 11, 1518. 10.3390/diagnostics11081518 PMC8391570 34441451

[B36] Moisi M. I. Rus M. Bungau S. Zaha D. C. Uivarosan D. Fratila O. (2020). Acute coronary syndromes in chronic kidney disease: Clinical and therapeutic characteristics. Medicina 56, 118. 10.3390/medicina56030118 PMC7143276 32182690

[B37] Moreno J. A. Moreno S. Rubio-Navarro A. Gómez-Guerrero C. Ortiz A. Egido J. (2014). Role of chemokines in proteinuric kidney disorders. Expert Rev. Mol. Med. 16, e3. 10.1017/erm.2014.3 24534600

[B38] Morii T. Fujita H. Narita T. Shimotomai T. Fujishima H. Yoshioka N. (2003). Association of monocyte chemoattractant protein-1 with renal tubular damage in diabetic nephropathy. J. Diabetes Complicat. 17, 11–15. 10.1016/s1056-8727(02)00176-9 12505750

[B39] Mottl A. K. Kwon K. S. Mauer M. Mayer-Davis E. J. Hogan S. L. Kshirsagar A. V. (2013). Normoalbuminuric diabetic kidney disease in the U.S. population. J. Diabetes Complicat. 27, 123–127. 10.1016/j.jdiacomp.2012.09.010 PMC4594950 23182925

[B40] Nakagawa T. Tanabe K. Croker B. P. Johnson R. J. Grant M. B. Kosugi T. (2011). Endothelial dysfunction as a potential contributor in diabetic nephropathy. Nat. Rev. Nephrol. 7, 36–44. 10.1038/nrneph.2010.152 21045790 PMC3653134

[B41] Nakajima K. Tanaka Y. Nomiyama T. Ogihara T. Ikeda F. Kanno R. (2003). RANTES promoter genotype is associated with diabetic nephropathy in type 2 diabetic subjects. Diabetes Care 26, 892–898. 10.2337/diacare.26.3.892 12610055

[B42] Navarro-González J. F. Mora-Fernández C. Muros de Fuentes M. García-Pérez J. (2011). Inflammatory molecules and pathways in the pathogenesis of diabetic nephropathy. Nat. Rev. Nephrol. 7, 327–340. 10.1038/nrneph.2011.51 21537349

[B43] Nikolic-Paterson D. J. (2016). Cathepsin S-dependent protease-activated receptor-2 activation: A new mechanism of endothelial dysfunction. J. Am. Soc. Nephrol. 27, 1577–1579. 10.1681/ASN.2015101162 26590253 PMC4884124

[B44] Papadopoulou-Marketou N. Kanaka-Gantenbein C. Marketos N. Chrousos G. P. Papassotiriou I. (2017). Biomarkers of diabetic nephropathy: A 2017 update. Crit. Rev. Clin. Lab. Sci. 54, 326–342. 10.1080/10408363.2017.1377682 28956668

[B45] Park J. Ryu D. R. Li J. J. Jung D. S. Kwak S. J. Lee S. H. (2008). MCP-1/CCR2 system is involved in high glucose-induced fibronectin and type IV collagen expression in cultured mesangial cells. Am. J. Physiol. Ren. Physiol. 295, F749–F757. 10.1152/ajprenal.00547.2007 18579703

[B46] Pérez-Morales R. E. Del Pino M. D. Valdivielso J. M. Ortiz A. Mora-Fernández C. Navarro-González J. F. (2019). Inflammation in diabetic kidney disease. Nephron 143, 12–16. 10.1159/000493278 30273931

[B47] Pichler R. Afkarian M. Dieter B. P. Tuttle K. R. (2017). Immunity and inflammation in diabetic kidney disease: Translating mechanisms to biomarkers and treatment targets. Am. J. Physiol. Ren. Physiol. 312, F716–F731. 10.1152/ajprenal.00314.2016 PMC6109808 27558558

[B48] Ricardo S. D. van Goor H. Eddy A. A. (2008). Macrophage diversity in renal injury and repair. J. Clin. Invest. 118, 3522–3530. 10.1172/JCI36150 18982158 PMC2575702

[B49] Samsu N. (2021). Diabetic nephropathy: Challenges in pathogenesis, diagnosis, and treatment. Biomed. Res. Int. 2021, 1497449. 10.1155/2021/1497449 34307650 PMC8285185

[B50] Satirapoj B. (2018). Tubulointerstitial biomarkers for diabetic nephropathy. J. Diabetes Res. 2018, 2852398. 10.1155/2018/2852398 29577044 PMC5822931

[B51] Schmid H. Boucherot A. Yasuda Y. Henger A. Brunner B. Eichinger F. (2006). Modular activation of nuclear factor-kappaB transcriptional programs in human diabetic nephropathy. Diabetes 55, 2993–3003. 10.2337/db06-0477 17065335

[B52] Seok S. J. Lee E. S. Kim G. T. Hyun M. Lee J. H. Chen S. (2013). Blockade of CCL2/CCR2 signalling ameliorates diabetic nephropathy in db/db mice. Nephrol. Dial. Transpl. 28, 1700–1710. 10.1093/ndt/gfs555 23794669

[B53] Shannon P. Markiel A. Ozier O. Baliga N. S. Wang J. T. Ramage D. (2003). Cytoscape: A software environment for integrated models of biomolecular interaction networks. Genome Res. 13, 2498–2504. 10.1101/gr.1239303 14597658 PMC403769

[B54] Su W. Zhao Y. Wei Y. Zhang X. Ji J. Yang S. (2021). Exploring the pathogenesis of psoriasis complicated with atherosclerosis *via* microarray data analysis. Front. Immunol. 12, 667690. 10.3389/fimmu.2021.667690 34122426 PMC8190392

[B55] Szklarczyk D. Morris J. H. Cook H. Kuhn M. Wyder S. Simonovic M. (2017). The STRING database in 2017: Quality-controlled protein-protein association networks, made broadly accessible. Nucleic Acids Res. 45, D362–D368. 10.1093/nar/gkw937 27924014 PMC5210637

[B56] Tan S. M. Ziemann M. Thallas-Bonke V. Snelson M. Kumar V. Laskowski A. (2020). Complement C5a induces renal injury in diabetic kidney disease by disrupting mitochondrial metabolic agility. Diabetes 69, 83–98. 10.2337/db19-0043 31624141

[B57] The Gene Ontology Consortium (2019). The gene Ontology resource: 20 years and still GOing strong. Nucleic Acids Res. 47, D330–D338. 10.1093/nar/gky1055 30395331 PMC6323945

[B58] Vaidya V. S. Niewczas M. A. Ficociello L. H. Johnson A. C. Collings F. B. Warram J. H. (2011). Regression of microalbuminuria in type 1 diabetes is associated with lower levels of urinary tubular injury biomarkers, kidney injury molecule-1, and N-acetyl-β-D-glucosaminidase. Kidney Int. 79, 464–470. 10.1038/ki.2010.404 20980978 PMC3033751

[B59] Van Gorp H. Delputte P. L. Nauwynck H. J. (2010). Scavenger receptor CD163, a Jack-of-all-trades and potential target for cell-directed therapy. Mol. Immunol. 47, 1650–1660. 10.1016/j.molimm.2010.02.008 20299103

[B60] Vesey D. A. Hooper J. D. Gobe G. C. Johnson D. W. (2007). Potential physiological and pathophysiological roles for protease-activated receptor-2 in the kidney. Nephrol. Carlt. 12, 36–43. 10.1111/j.1440-1797.2006.00746.x 17295659

[B61] Wang X. Shaw S. Amiri F. Eaton D. C. Marrero M. B. (2002). Inhibition of the Jak/STAT signaling pathway prevents the high glucose-induced increase in tgf-beta and fibronectin synthesis in mesangial cells. Diabetes 51, 3505–3509. 10.2337/diabetes.51.12.3505 12453907

[B62] Wilkinson R. D. Williams R. Scott C. J. Burden R. E. (2015). Cathepsin S: Therapeutic, diagnostic, and prognostic potential. Biol. Chem. 396, 867–882. 10.1515/hsz-2015-0114 25872877

[B63] Wolf G. Aberle S. Thaiss F. Nelson P. J. Krensky A. M. Neilson E. G. (1993). TNF alpha induces expression of the chemoattractant cytokine RANTES in cultured mouse mesangial cells. Kidney Int. 44, 795–804. 10.1038/ki.1993.314 7505037

[B64] Wolf G. Ziyadeh F. N. Thaiss F. Tomaszewski J. Caron R. J. Wenzel U. (1997). Angiotensin II stimulates expression of the chemokine RANTES in rat glomerular endothelial cells. Role of the angiotensin type 2 receptor. J. Clin. Invest. 100, 1047–1058. 10.1172/JCI119615 9276721 PMC508279

[B65] Woroniecka K. I. Park A. S. Mohtat D. Thomas D. B. Pullman J. M. Susztak K. (2011). Transcriptome analysis of human diabetic kidney disease. Diabetes 60, 2354–2369. 10.2337/db10-1181 21752957 PMC3161334

[B66] Xu Q. Li B. Wang Y. Wang C. Feng S. Xue L. (2021). Identification of VCAN as hub gene for diabetic kidney disease immune injury using integrated bioinformatics analysis. Front. Physiol. 12, 651690. 10.3389/fphys.2021.651690 34557107 PMC8454927

[B67] You H. Gao T. Cooper T. K. Brian Reeves W. Awad A. S. (2013). Macrophages directly mediate diabetic renal injury. Am. J. Physiol. Ren. Physiol. 305, F1719–F1727. 10.1152/ajprenal.00141.2013 PMC3882451 24173355

[B68] Zhang L. Long J. Jiang W. Shi Y. He X. Zhou Z. (2016). Trends in chronic kidney disease in China. N. Engl. J. Med. 375, 905–906. 10.1056/NEJMc1602469 27579659

